# PANX1 is a potential prognostic biomarker associated with immune infiltration in pancreatic adenocarcinoma: A pan-cancer analysis

**DOI:** 10.1080/19336950.2021.2004758

**Published:** 2021-11-19

**Authors:** Lingling Bao, Kai Sun, Xuede Zhang

**Affiliations:** a Department of Hematology and Oncology, Beilun District People’s Hospital, Ningbo, Zhejiang, China; b Liuzhou People’s Hospital, Liuzhou, Guangxi, China

**Keywords:** Pannexin 1, cancer, immune infiltration, prognosis, biomarker

## Abstract

Pannexin 1 (PANX1) channel is a critical ATP-releasing pathway that modulates tumor immunity, progression, and prognosis. However, the roles of PANX1 in different cancers remain unclear. We analyzed the expression of PANX1 in human pan-cancer in the Oncomine and GEPIA2.0 databases. The prognostic value of PANX1 expression was determined using Kaplan-Meier plotter and OncoLnc tools. The correlation between PANX1 and tumor-infiltrating immune cells was investigated using the TIMER 2.0. In addition, the relationship between PANX1 and immunomodulators was explored using TISIDB. Finally, gene set enrichment analysis (GSEA) was performed utilizing LinkedOmics. The results indicated that PANX1 was overexpressed in most cancers compared to normal tissues. The high expression of PANX1 was associated with poor prognosis in multiple tumors, especially in pancreatic adenocarcinoma (PAAD). In addition, PANX1 was correlated with a variety of immunomodulators, such as CD274, IL10, CD276, IL2RA, TAP1, and TAP2. PANX1 expression level was significantly related to infiltration of multiple immune cells in many cancers, including cancer associated fibroblast, macrophage, and neutrophil cells. Further analysis revealed that PANX1 was significantly associated with T cells CD8+ (rho = 0.524, *P* = 1.94e-13) and Myeloid dendritic cell (rho = 0.564, *P* = 9.45e-16). GSEA results showed that PANX1 was closely associated with leukocyte cell-cell adhesion, endoplasmic reticulum lumen, ECM-receptor interaction, and Focal adhesion pathways in PAAD. PANX1 expression was higher in pan-cancer samples than in normal tissues. The high expression of PANX1 was associated with poor outcome and immune infiltration in multiple cancers, especially in PAAD.

## Introduction

According to recent research, the pannexin 1 (PANX1) channel is an important ATP-releasing pathway [1–3]. Accumulating evidence also indicates that PANX1 is overexpressed in a variety of cancers and regulates the tumor immune microenvironment via ATP release channels [4,5]. Three members of the pannexin family are known: pannexin-1, pannexin −2, and pannexin −3 [6]. While PANX1 is widely expressed in a variety of human tissues, pannexin-2 and pannexin-3 are more abundant in the brain, skin, and bone [5]. Therefore, PANX1 may have a wide variety of biological effects on cancer development. Previous studies have reported that PANX1 promotes cell proliferation and tumorigenic properties in melanoma cells [7–9], testicular cancer [10], and breast cancer [11–13]. PANX1-blocking therapeutics may be an effective strategy for tumor therapy, as previously discussed [14]. However, research on the role of PANX1 in human pan-cancer is limited.

One of the major features of the tumor microenvironment (TME) is increased extracellular adenosine triphosphate (ATP), which is decreased in healthy tissues [15,16]. Extracellular ATP levels play essential roles in tumor progression via a variety of molecular mechanisms, including cell growth, cell differentiation, energy metabolism, and intercellular signaling [17,18]. Several studies have shown that extracellular ATP release regulated immunological responses by recruiting and activating immune cells [19–21].

The tumor microenvironment has become a focal point in cancer research in recent years. Substantial evidence demonstrates that TME plays a vital role in tumorigenesis, tumor growth, invasion, and migration. The TME is complex and consists of tumor cells, immune cells, fibroblasts, extracellular matrix (ECM), growth factors, and cellular metabolites [22]. Numerous studies have demonstrated that TME, particularly tumor immune cell infiltration, has a significant effect on tumor development and may be used to predict the prognosis of tumors [23,24]. However, the potential functions and mechanisms of PANX1 in tumor progression and tumor immunity remain unclear.

In this study, using an online database, we conducted a pan-cancer analysis to investigate the expression profiles and prognostic landscape of PANX1. Furthermore, we investigated the relationship between PANX1 and tumor-infiltrating immune cells in different tumors using Tumor Immune Estimation Resource (TIMER) and TISIDB (http://cis.hku.hk/TISIDB/). The findings showed that PANX1 may influence the development and prognosis of various tumors by regulating tumor immune cell infiltration in the TEM.

## Materials and methods

### Mutations and copy number alterations of PANX1

In this study, the cBioPortal online tool (http://www.cbioportal.org/) was used to investigate the genomic alterations of PANX1 in various cancers. PANX1 genomic alteration (mutation, structural variant, amplification, deep deletion, multiple alterations) from The Cancer Genome Atlas (TCGA) pan-cancer database was visualized using cBioPortal [25].

### PANX1 expression profiles

The oncomine database (www.oncomine.org) provides easy and user-friendly access to explore gene expression profiles based on the cancer microarray database [26]. We, therefore, investigated differential expression of PANX1 between various tumors and normal tissues using the oncomine database. The thresholds were set as *P*-value <0.01 and fold change ≥1.5. GEPIA2 (http://ualcan.path.uab.edu/c) is an online data-mining platform based on the TCGA and GTEx databases, that can be used to analyze gene expression in various cancers. In the present study, we also used GEPIA2 to investigate the expression profiles of PANX1 across pan-cancer. The cancer tissue data were obtained from the TCGA database. The matched normal tissues data was obtained from TCGA and GTEx databases. Four-way analysis of variance (ANOVA) was used to perform differential analysis using sex, age, ethnicity, and disease state (tumor or normal) as variables. The *P*-value <0.01 and fold change ≥1.5 were set as thresholds for significant differential expression.

### Pan-cancer survival analysis

In this study, we performed a pan-cancer survival analysis of PANX1 using two survival analysis tools (the Kaplan Meier plotter and OncoLnc). The Kaplan Meier plotter is an online tool that is widely used to perform survival analysis across 21 cancer types [27]. The website provides a comprehensive prognosis analysis using the Kaplan-Meier approach based on multiple databases, including GEO, EGA, and TCGA. The *p*-value obtained exclusively by Kaplan-Meier analysis may be deceptive, and it may be more useful to examine the relative strength of the correlation [28]. The OncoLnc provided a more thorough survival analysis with Cox regression based on TCGA pan-cancer data, and a model was constructed that included gene expression, sex, age, and grade or histology as multivariates whenever possible for each cancer [28].

### Immune infiltrates analysis

TISIDB is an online platform for tumor and immune system interactions, that integrates multiple heterogeneous data types. TISIDB enables researchers to investigate the relationships between selected genes and immune features in various tumors [29]. In the present study, we explored the associations between PANX1 and immunomodulators in pan-cancer.

TIMER 2.0 (http://timer.cistrome.org/) is an interactive web platform used to perform a systematic analysis of immune infiltrates and gene expression across different cancer types [30]. The website allows researchers to select genes of interest and examine the relationship between immune infiltrates and gene expression in different cancer types. TIMER2.0 includes six different state-of-the-art algorithms, TIMER [31], xCell [32], MCP-COUNTER [33], CIBERSORT [34], EPIC [35], and QUANTISEQ [36], to assess the correlation between immune infiltration and gene expression across various tumor types.

### Enrichment analysis

LinkedOmics is a user-friendly publicly accessible bioinformatics website that offers multi-omics analysis across 32 TCGA cancer types [37]. The LinkFinder module was used to investigate positively and negatively significantly correlated genes of PANX1 in selected cancers. The LinkInterpreter module uses gene set enrichment analysis (GSEA) to perform GO and KEGG pathway analyses of the significantly correlated genes. GO terms included those related to biological process (BP), cellular component (CC), and molecular function (MF). The following were the parameters for the enrichment analysis, the category must have had a minimum of three genes, the category’s maximum number of genes was 2000, the significance level was top 25, and the number of permutations was 500. FDR < 0.25 and *P* < 0.05 were considered statistically significant.

### Statistical analysis

Oncomine and GEPIA2 were used to examine differential expression between cancer tissues and normal tissues. Kaplan-Meier survival and Cox regression analyses were used to conduct the survival analyses. Spearman’s correlation was used to examine the relationships between tumor-immune feather and PANX1 expression in the TIMER 2.0 and TISIDB datasets. r ≥ 0.5 was considered a strong correlation, 0.5 > r ≥ 0.3 was considered moderate correlation, and 0.3 > r ≥ 0.1 was considered as weak correlation [38]. GSEA was used to perform enrichment analyses. FDR < 0.25 and *P* < 0.05 were considered statistically significant.

## Results

### PANX1 genes alterations

**Figure 1.**
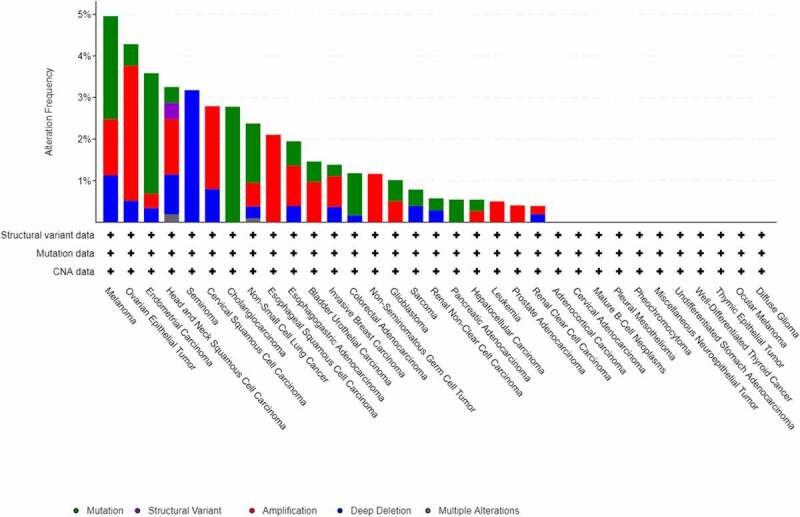
PANX1 mutation frequency in human cancers according to data on the cBioPortal database

Using the cBioPortal web tool, we investigated PANX1 gene alterations in 33 cancer types and 10,953 patients. As a result, 179 (1.6%) of the 10,953 cancer patients had PANX1 gene alterations. The top three genes with the highest rates of PANX1 gene alterations were present in melanoma (4.95% of 444 cases), ovarian epithelial tumor (4.28% of 584 cases), and endometrial carcinoma (5.58% of 586 cases) (Figure 1).

### PANX1 expression profiles in human cancers

**Figure 2.**
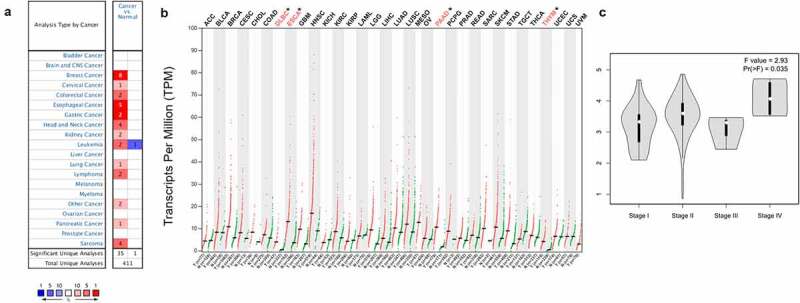
PANX1 expression levels in different types of human cancers. (a) Upregulated and downregulated PANX1 expression in datasets of various cancers in the Oncomine database. (b) PANX1 expression levels in different tumor types in the GEPIA2 database (*P < 0.05). (c) Expression of PANX1 in different clinical stages of PAAD

Oncomine and GEPIA 2.0 databases were used to investigate the levels of PANX1 mRNA expression levels in various human cancers and adjacent normal tissues. PANX1 was shown to be highly expressed in the vast majority of tumors in the Oncomine database, including breast cancer, cervical cancer, colorectal cancer, esophageal cancer (ESCA), gastric cancer, head and neck cancer, kidney cancer, lymphoma, leukemia, lung cancer, pancreatic cancer, and sarcoma. However, PANX1 gene expression findings in leukemia are inconsistent. PANX1 is upregulated in two datasets and down-regulated in one dataset. (Figure 2(a)). Figure 2(b) depicts differential expression patterns of PANX1 in tumors and paired normal tissues from the GEPIA database. PANX1 was shown to be overexpressed in diffuse large B-cell lymphoma (DLBC), ESCA, Pancreatic adenocarcinoma (PAAD), and Thymoma (THYM) compared with paired normal tissues. We investigated the relationship between PANX1 expression and cancer stages in ESCA, DLBC, and PAAD, using Oncomine and GEPIA 2.0. PANX1 expression was shown to be elevated in stage IV PAAD patients (P = 0.035) (Figure 2(c)).

### PANX1 prognostic value analysis in human cancers

**Figure 3.**
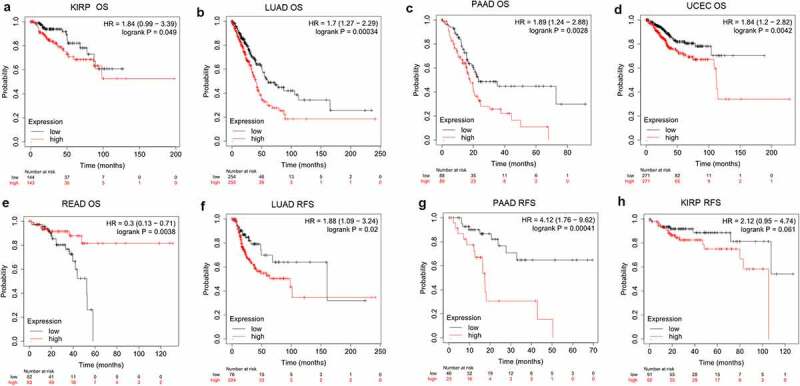
The association of PANX1 expression with prognosis of various cancers as determined by Kaplan Meier plotter analysis. (a-e), Determination of the OS of five cancer types (KIRP, LUAD, PAAD, UCEC, and READ) by Kaplan–Meier analysis. (f-h), Analysis of RFS in three cancer types (KIRP, LUAD, and PAAD) by Kaplan–Meier plotter

To investigate the prognostic predictive ability of PANX1 in human cancers, we performed univariate survival analysis to determine the association between PANX1 expression and the overall survival (OS) of each cancer. Based on the median value of PANX1 mRNA in individual tumors, cancer patients were classified into high and low expression groups. The Kaplan-Meier curve and log-rank value were calculated using Kaplan Meier plotter online tools. Figure 3 shows the Kaplan-Meier survival analysis findings for OS. The survival curves revealed that high PANX1 expression may be associated with a worse prognosis in patients with kidney renal papillary cell carcinoma (KIRP) (*P* = 0.049, HR = 1.84, 95% CI = 0.99–3.39), lung adenocarcinoma (LUAD) (*P* = 0.00034, HR = 1.7, 95%CI = 1.27–2.29), PAAD (*P* = 0.0028, HR = 1.89, 95%CI = 1.27–2.29), and uterine corpus endometrial carcinoma (UCEC) (*P* = 0.0042, HR = 1.84, 95%CI = 1.2–2.82). High PANX1 expression, on the other hand, predicted a better prognosis for patients with rectum adenocarcinoma (READ) (*P* = 0.0038, HR = 0.3, 95% CI = 0.13–0.71).

To further determine if PANX1 expression was an independent prognostic factor for human cancers, multivariate COX analysis of OS was performed in OncoLnc database. As shown in Table 1, high PANX1 expression increased the risk of death of KIRP (Cox Coefficient = 0.641; *P* = 4.20e-04), LUAD (Cox Coefficient = 0.221; *P* = 3.30e-03), PAAD (Cox Coefficient = 0.275; *P* = 9.00e-03), bladder Urothelial Carcinoma (BLCA) (Cox Coefficient = 0.193; *P* = 0.017), breast invasive carcinoma (BRCA) (Cox Coefficient = 0.216; *P* = 0.026), cervical squamous cell carcinoma, and endocervical adenocarcinoma (CESC) (Cox Coefficient = 0.3; *P* = 0.028) patients.

**Table 1.** Correlation of PANX1mRNA expression and clinical prognosis in human cancers by OncoLnc database

Cancer	Cox Coefficient	P-Value	FDR Corrected	Median Expression	Mean Expression
KIRP	0.641	4.20e-04*	5.71e-03	234.19	265.4
LUAD	0.221	3.30e-03*	4.70e-02	503.93	550.11
PAAD	0.275	9.00e-03*	7.32e-02	526.6	553.18
BLCA	0.193	1.70e-02*	1.35e-01	431.59	531.13
BRCA	0.216	2.60e-02*	3.00e-01	471.23	508.85
CESC	0.3	2.80e-02*	2.57e-01	406.15	492.86
COAD	0.191	5.90e-02	4.28e-01	327.27	340.21
OV	0.115	1.00e-01	6.69e-01	355.88	367.73
GBM	0.139	1.20e-01	7.93e-01	400.71	415.53
LUSC	0.097	1.60e-01	6.72e-01	588.03	660.41
UCEC	0.105	3.30e-01	9.92e-01	309.16	330.21
ESCA	−0.135	3.80e-01	9.77e-01	555.49	700.77
LGG	−0.073	3.90e-01	4.96e-01	385.25	403.62
LIHC	0.07	4.30e-01	6.78e-01	415.59	516.18
STAD	0.069	4.30e-01	7.73e-01	464.89	485.42
KIRC	−0.062	4.40e-01	5.69e-01	408.55	462.66
READ	−0.192	4.40e-01	9.60e-01	309.66	326.49
LAML	0.076	4.60e-01	7.87e-01	294.45	306.14
HNSC	0.045	5.20e-01	7.93e-01	894.69	1049.89
SKCM	0.024	7.30e-01	8.45e-01	504.48	569.6
SARC	−0.033	7.40e-01	8.88e-01	476.44	526.14

In addition to the OncoLnc database and Kaplan Meier plotter findings, KIRP, LUAD, and PAAD were selected for further analysis of recurrence-free survival (RFS). PANX1 was also a predictor of worse RFS for LUAD (*P* = 0.02, HR = 1.88, 95% CI = 1.09–3.24) and PAAD (*P* = 0.00041, HR = 4.12, 95% CI = 1.76–9.62).

### Relationship between immunomodulators and PANX1

**Figure 4.**
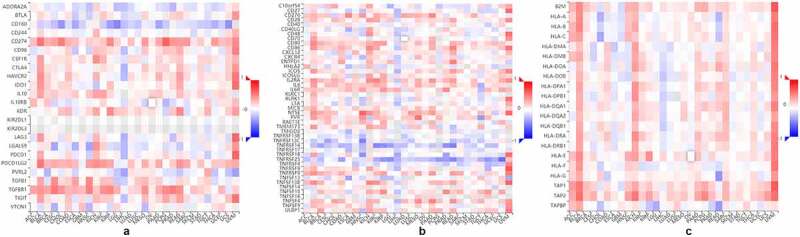
Association of PANX1 with immunomodulators. (a) Association of PANX1 expression with immuneinhibitors. (b) Association of immunostimulators with PANX1 expression (c) Correlation between MHC molecules and PANX1 expression. Red and blue cells show positive and negative correlations, respectively. The intensity of color is proportional to the strength of the correlation

Because ATP is a well-known immunomodulator, we further investigated the relationship between PANX1 expression and immunomodulators in human cancers. As Figure 4(a) shown, PANX1 was positively correlated with several well-known immunoinhibitors (including CD274, IL10, PDCD1LG2, and TGFBR1) in most cancers. CD160 was implicated in a variety of immunological responses, including T cell inhibition and natural killer cell activation [39]. In the present study, PANX1 was shown to have a negative correlation with CD160 in various tumors, such as LGG (rho = −0.480, P < 2.2e-16), KIRP (rho = −0.371, P = 9e-11) and cholangiocarcinoma (CHOL) (rho = −0.41, P = 0.0137). Figure 4(b) shows the correlations between PANX1 expression levels and immunostimulants in a variety of cancers. For example, PANX1 correlated positively with CD276 in ESCA (rho = 0.461, P = 5.45e-11), head and neck squamous cell carcinoma (HNSC) (rho = 0.391, P < 2.2e-16), and LUAD (rho = 0.348, P = 4.2e-16). There was also a positive correlation between PANX1 expression and IL2RA in BLCA (rho = 0.429, P < 2.2e-16), KIRC (rho = 0.482, P < 2.2e-16), and KICH (rho = 0.451, P < 0.01). However, PANX1 exhibited a negative correlation with TNFRSF25 in uterine carcinosarcoma (rho = – 0.578, P = 3.87e-06), READ (rho = −0.459, P = 6.88e-10), and LGG (rho = −0.44, P = 4.2e-16). PANX1 showed positive correlations with TAP1 and TAP2 in several cancer types, including BLCA, KICH, adrenocortical carcinoma (ACC), and uveal melanoma (UVM) for the MHC molecule (Figure 4(c)). PANX1 expression was also found to be positively correlated with the majority of MHC molecules in BLAC, KICH, PAAD, sarcoma, and UVM.

According to the gene expression and prognosis analysis, we found that the expression status and predicted values of PANX1 were inconsistent in different cancers across different databases. PANX1 overexpression in PAAD was simultaneously confirmed in two databases (Oncomine and GEPIA 2.0). Furthermore, the two databases (OncoLnc and Kaplan Meier plotter) demonstrated that PANX1 is an unfavorable prognostic biomarker in PAAD. Thus, PAAD was selected as a representative cancer type for subsequent analysis.

**Figure 5.**
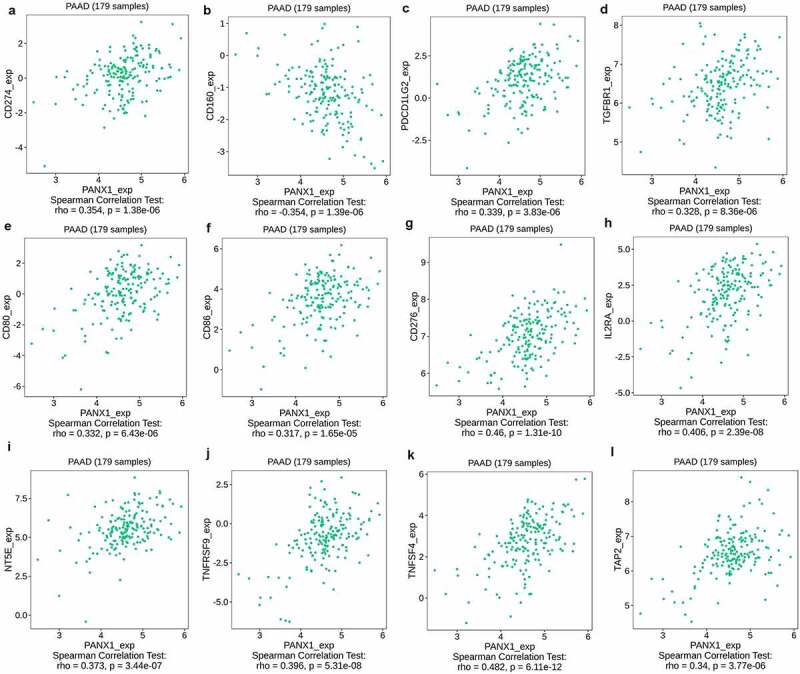
Association of PANX1 with immunomodulators in PAAD. (a-d) Immuneinhibitors that were moderately (0.5 > r ≥ 0.3) or strongly (r ≥ 0.5) correlated with PANX1 in PAAD. (e-k) immunostimulators that were moderately or strongly correlated with PANX1 in PAAD. (l) MHC molecules that showed moderate or strong correlation with PANX1 in PAAD

PANX1 expression was found to be significantly correlated with immunoinhibitors such as CD274 (rho = 0.354, *P* = 1.38e-06), CD160 (rho = −0.354, *P* = 1.39e-06), PDCD1LG2 (rho = 0.339, *P* = 3.83e-06), and TIFBR1 (rho = 0.328, *P* = 3.83e-06) (Figure 5(a-d)). PANX1 expression was also closely related to immunostimulators, such as CD80 (rho = 0.332, P = 6.43e-06), CD86 (rho = 0.317, P = 1.65e-06), CD276 (rho = 0.46, p = 1.31e-10), IL2RA (rho = 0.406, P = 2.39e-08), NT5E (rho = 0.373, P = 3.44e-07), TNFRSF9 (rho = 0.396, P = 5.31e-08), and TNFSF4 (rho = 0.482, P = 6.11e-12) (Figure 5(e-k)). Furthermore, PANX1 expression was also associated with MHC molecule, such as TAP2 (rho = 0.34, P = 3.77e-06) (Figure 5(l)).

### Relationship between PANX1 expression and immune infiltration in human cancers

The tumor microenvironment comprises stromal cells, tumor cells, and immune infiltrating cells. TIMER2.0 was used to determine the relationship between PANX1 expression and various infiltrating immune cells in diverse cancer types, to better understand the role in tumor immune infiltration. TIMER2.0 included six algorithms, TIMER, xCell, MCP-COUNTER, CIBERSORT, EPIC, and QUANTISEQ, which computed immune infiltration estimates.

**Figure 6.**
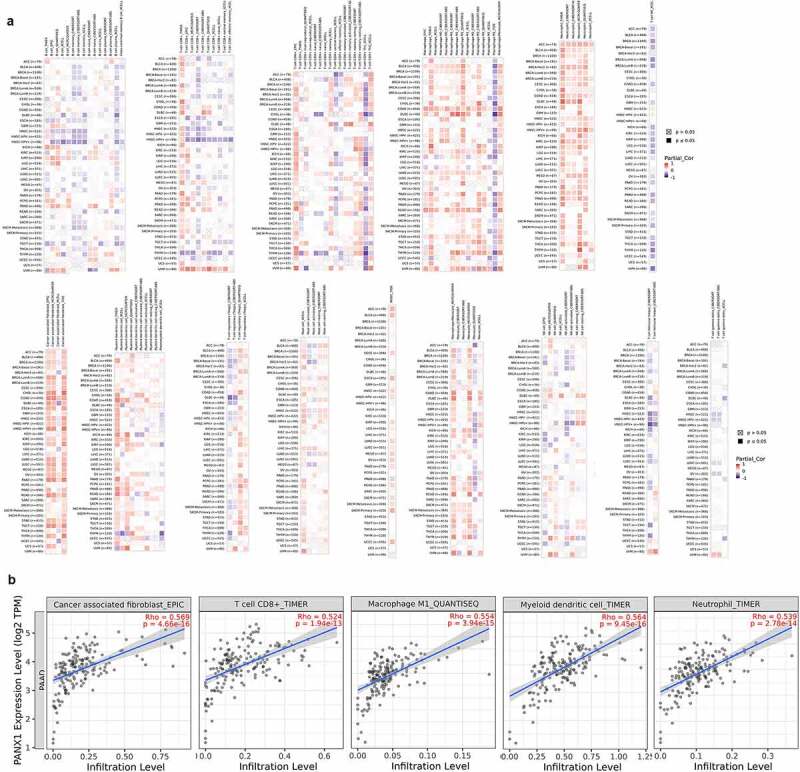
Association of PANX1 expression with immune infiltration. (a) Association of PANX1 expression with immune infiltration as determined using by different algorithms in TIMER 2.0. (b) PANX1 expression was strongly related to infiltration level of various immune cells in PAAD (r ≥ 0.5)

According to multiple algorithms, the expression of PANX1 was positively correlated with cancer-associated fibroblast (CAF), macrophage, and neutrophils in most cancer types as shown in Figure 6(a). PANX1 expression, on the other hand, was negatively correlated with T cell NK. There was a wide range of variance in the relationship between gene expression and immune infiltrating level according to different algorithms. PANX1 expression, for example, was positively correlated with T cell CD8+ infiltration when TIMER was used, but negatively correlated with T cell CD8+ when CIBERSORT was used. MCP-COUNTER revealed a strong correlation between PANX1 expression and monocytes, but a negative correlation when QUANTISEQ was used. In the majority of cancer types, PANX1 expression demonstrated a significant positive association with T cell regulatory using QUANTISEQ, but a negative correlation using CIBERSORT. Additionally, we discovered a significant positive correlation between PANX1 expression and myeloid dendritic cell (DC) using TIMER, but not with other methods. According to multiple algorithms, the relationships between PANX1 expression and B cells in various cancers were complex and diverse.

Tumor immune infiltrates vary greatly among cancer types, possibly because the heterogeneity of immune infiltration is a feature intrinsic to tumor cells. Six algorithms found a significant positive relationship between PANX1 expression and T cell CD8+ in UVM. Almost all algorithms, with the exception of the TIMER method, found a negative correlation between PANX1 expression and T cell CD8+ in HNSC-HPV+ types.

We then conducted a more in-depth investigation of the relationship between PANX1 expression and immune infiltration in PAAD. PANX1 was shown to have a positive correlation with the majority of infiltrating immune cells, including T cells CD8+_TIMER (rho = 0.524, *P* = 1.94e-13), Neutrophil-TIMER (rho = 0.539, *P* = 2.78e-14), MacrophageM1_QUANTISEQ (rho = 0.5549, *P* = 3.94e-15), Myeloid dendritic cell_TIMER (rho = 0.564, *P* = 9.45e-16), and CAF_EPIC (rho = 0.569, *P* = 4.66e-16) (Figure 6(b)).

### Enrichment analyses of genes co-expressed with PANX1 in PAAD

**Figure 7.**
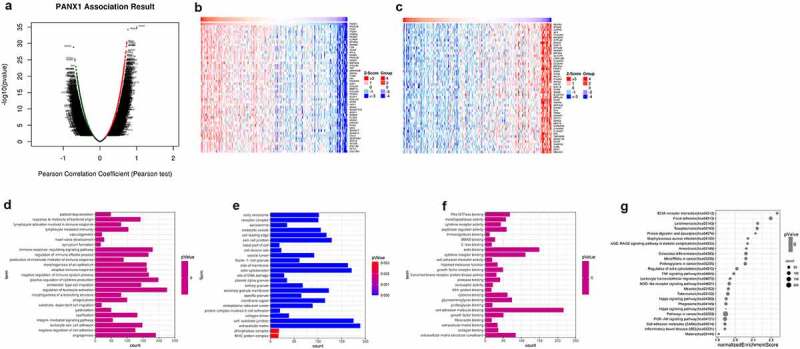
Function and pathway enrichment analyses for genes co-expressed with PANX1 in PAAD. (a) Volcano plot showing genes co-expressed with PANX1 in PAAD. (b) A heatmap of top 50 genes most positively associated with PANX1. (c) A heatmap of top 50 genes most negatively associated with PANX1. (d) The top 25 positively correlated GO terms in the BP category. (e) The top 25 positively correlated GO terms in the CC category. (f) The top 25 positively correlated GO terms in the MF category. (g) Top 25 positively correlated KEGG pathways. Red indicates positively correlated genes, and green indicates negatively correlated genes

**Figure 8.**
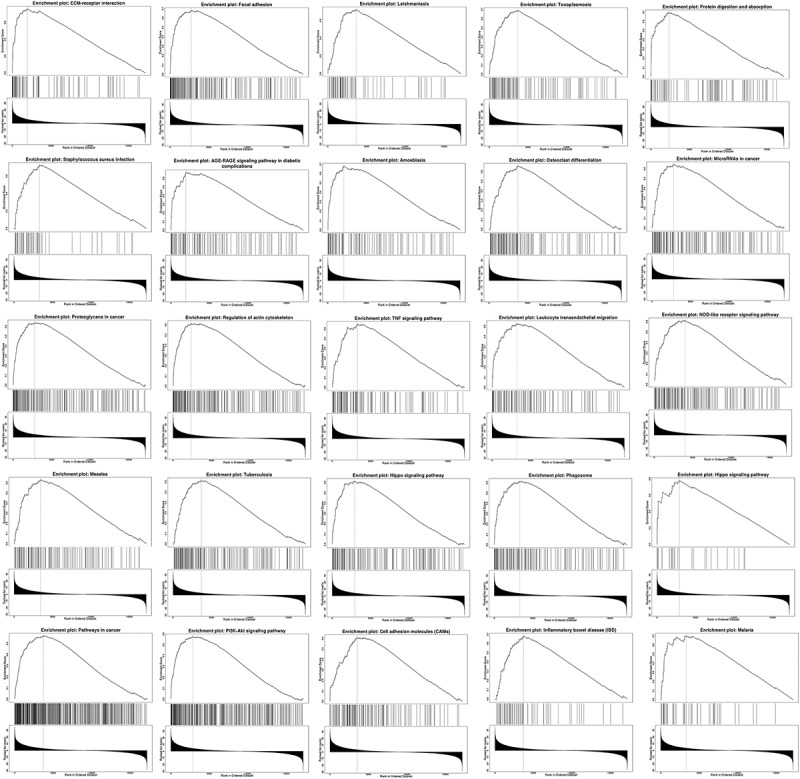
Top 25 positively correlated GSEA enrichment plots

Linkedomics was used to perform enrichment analysis of co-expression genes associated with PANX1 in PAAD. As shown in the volcano plot (Figure 7(a)), 3233 positively associated genes (red) and 2954 negatively associated genes (green) were identified in PAAD (*P* < 0.05, FDR < 0.01). A heat map was used to display the top 50 positively and negatively correlated genes in PAAD (Figure 7(b,c)). GO BP analysis showed that co-expressed genes were involved in angiogenesis, negative regulation of cell adhesion, leukocyte cell-cell adhesion, integrin-mediated signaling pathway, and ossification. GO CC analysis showed that these genes were mainly enriched in extracellular matrix cell-substrate junction, collagen trimer, a protein complex involved in cell adhesion, and endoplasmic reticulum lumen. GO MF analysis showed that these genes were predominantly enriched in extracellular matrix structural constituent, collagen binding, extracellular matrix binding, fibronectin-binding, and growth factor binding (Figure 7(d-f)). KEGG pathway analysis showed that the co-expressed genes were enriched in ECM-receptor interaction, focal adhesion, leishmaniasis, toxoplasmosis, and protein digestion and absorption in PAAD (Figure 7(g)). Figure 8 shows the detailed GSEA enrichment plots.

## Discussion

Several studies published in recent years have found that extracellular ATP, an important biochemical component of TME, is implicated in immune cell recruitment and activation, which leads to tumor proliferation and metastasis [40–45]. PANX1 is a key ATP release channel that may be activated or deactivated by a variety of events, including the P2X7 receptor, intracellular Ca2+, extracellular K+, and mechanical stimulation [46–49]. Currently, only a modest amount of research has been conducted on the involvement of PANX1 in tumor progression and immune response [7,12,50]. However, the roles of PANX1 in tumor pathogenesis and immune infiltration remain unknown. Therefore, we performed a pan-cancer analysis of PANX1 using multiple databases to evaluate the features of gene expression, prognosis, and tumor immunity.

In this study, we used the cBioPortal website to analyze PANX1 gene alterations in 33 cancer types. The result showed that melanoma had the highest frequency of mutations. A recent study also found that the Y150F PANX1 mutation inhibited phosphorylation, N-glycosylation, as well as affecting the large-pore channel structure and function of PANX1 in melanoma [51]. A somatic nonsense mutation, Q89*, encoding a truncated form of PANX1 protein, was similarly shown to be enriched in highly metastatic breast cancer cells. The truncated Q89* protein augmented ATP release and enhanced breast cancer cell metastasis [13]. These findings showed that PANX1 mutations may affect protein function and contribute to tumor progression.

In this study, we investigated PANX1 expression levels and prognostic landscape in various cancer. Previous studies showed that PANX1 was overexpressed in hepatocellular carcinoma (HCC) [52], gastric cancer cells [53], and melanoma cells [7]. However, the expression patterns of PANX1 in other tumors are unknown. Therefore, we investigated the levels of PANX1 mRNA expression in various cancer types and found that PANX1 expression was significantly upregulated in many cancers, including ESCA, PAAD, and stomach adenocarcinoma (STAD). The findings suggested that PANX1 may play an important in tumorigenesis and cancer progression. Accumulating evidence suggests that PANX1 may enhance tumor proliferation, invasion, and metastasis by regulating extracellular ATP concentration in the TME. By activating P2 receptors, extracellular ATP increases tumor cell survival and proliferation [54,55]. A recent study demonstrated that the ATP activated AKT pathway through the P2X7 receptor promotes breast cancer cell invasion and migration [56]. These findings showed that PANX1 is an oncogene that plays a vital role in tumor invasion and metastasis. Only a few studies have been conducted to determine the PANX1’s prognostic power in cancer. A previous study reported that high PANX1 expression predicts a poor prognosis of HCC [52]. In the present study, the Kaplan Meier plotter results showed that high PANX1 expression was associated with a poor prognosis in patients with KIRP, LUAD, UCEC, and PAAD. However, our findings from the OncoLnc database showed that high PANX1 expression may be associated with a poor prognosis in patients with KIRP, LUAD, PAAD, BLCA, BRCA, and CESC. These findings suggest that PANX1 may predict poor OS in patients with KIRP, LUAD, and PAAD.

Another significant finding from this study is that PANX1 expression correlates with certain immunomodulators in a variety of cancers. PANX1 was shown to be positively associated with some immune inhibitors such as CD274, PDCD1LG2, and TGFBR1 in a variety of cancers. In various cancer types, programmed cell death-ligand 1 (PD-L1; encoded by the CD274 gene) and programmed cell death 1 ligand 2 (PD-L2, CD273, encoded by the PDCD1LG2) suppressed T cell activation and facilitated immune evasion by binding to PD-1 on lymphocytes [57,58]. Transforming growth factor (TGF)-β is thought to inhibit the activity of Th1 helper and cytotoxic T cell responses in TME and promote tumor immune evasion by binding to TGF-β receptor (TGFBR) [59,60]. Furthermore, there is ample evidence that TGF-β signaling exerts immunosuppressive roles by inducing T regulatory phenotype and suppressing NK cells and DCs [61]. The present findings were consistent with those of the previous study. PANX1 may promote tumor progression and immune suppression by co-expression of immunoinhibitors, such as CD274, PDCD1LG2, and TGFBR. PANX1 positive correlation with some immunostimulators such as CD276 and IL2RA seems surprising. CD276 (B7-H3) was originally thought to be an immunostimulatory molecule that regulated immune response. Recent research, however, suggests that it also plays an inhibitory role on T-cells, which contributes to tumor cell immune evasion [62]. IL2RA (CD25) was found to be highly expressed in CD4+ and CD8 + T cells. Subsequently, it was discovered that the vast majority of Tregs expressed high levels of CD25 [63]. According to these findings, IL2RA may play a variety of roles in tumor immune response. The present study also showed that PANX1 exhibited a negative correlation with TNFRSF14 and TNFRSF25 in a variety of tumors. The two TNFR superfamily genes promoted CD4+ and CD8 + T cell survival and play a critical role in antitumor immunity [64]. PANX1 expression was also shown to be positively correlated with certain MHC molecules in multiple malignancies. TAP1 and TAP2 are members of the ATP-binding cassette (ABC) transporter protein family that play essential roles in adaptive immunity by assisting antigen loading onto MHC I molecules and presentation to cytotoxic T lymphocytes [65]. The findings showed that PANX1 may be regulated by immunomodulators in tumors.

Immunotherapy has developed into a research hotspot in recent years. Immune cells infiltration levels are considered as critical determinants of immunotherapy response. PANX1 expressions were found to be positively correlated with a variety of immune infiltrating cells, including neutrophils, CAF, macrophages, MDSC, and monocytes in various cancers, such as PAAD, COAD, LUAD, READ, and UVM. Neutrophils have established themselves as a key component of TAM. Most studies have reported that neutrophils exert tumor-promoting functions, including angiogenesis, proliferation, extracellular matrix remodeling, epithelial-mesenchymal transition (EMT), and immunosuppression [66]. Numerous studies have also shown that macrophages play a critical role in tumor progression and metastasis, stimulating angiogenesis, promoting tumor cell survival, and suppressing antitumor immunity [67]. The present study indicated that PANX1 may contribute to immunosuppression by inducing neutrophil and macrophage infiltration in TME. The mechanism by which PANX1 affects the immune response to cancer is complex. Previous studies revealed that PANX1 is an essential ATP release channel. P2 purinergic receptors (P2Rs), which are distinct from P2X7 receptors in that they operate as extracellular ATP activating receptors, are highly expressed in a variety of immune cells. Extracellular ATP works through P2Rs to trigger recruitment and activation of various immune cells, including CD8+ and CD4 + T cells, Tregs, tumor-associated macrophages (TAMs), myeloid-derived suppressor cells (MDSCs), neutrophils, macrophages, and dendritic cells (DCs) [68]. This partially explains why PANX1 expression was associated with immune cell infiltration in TEM.

We further examined the relationship between PANX1 expression and the infiltrating immune cells in PAAD. PANX1 expression was found to be positively correlated with CD4+ Th2 and negatively correlated with CD4+ Th1 cells in PAAD. Th1 cells are generally considered favorable to induce an efficient antitumor immune response via the recruitment and enhancement of CD8 T cells [69]. High levels of Th1 cell infiltration in the TME have been associated with a good prognosis in various cancers, including the brain [70], colorectal [71], and ovarian cancers [72]. Many studies have reported that Th2 cells play an essential role in suppressing the antitumor immune response by releasing cytokines such as IL-4, IL-5, IL-6, IL-10, and IL-13 [73,74]. Several studies have reported that Th2 cells infiltration is associated with poor outcomes in ovarian [75] and gastric cancers [76]. Consistent with previous studies [77,78], our findings showed that elevated levels of Th2 cells infiltration and decreased levels of Th1 infiltration were associated with poor prognosis in PAAD patients. Treg infiltration has been observed in a variety of tumor tissues, suppressing effector T cell activity and promoting tumor progression and metastasis [79–81]. Treg infiltration was shown to be significantly associated with a poor prognosis in the lung [82], PAAD [83], and renal cancer patients [84]. PANX1 expression was shown to be positively associated with Tregs infiltration levels in PAAD, LUAD, UVM, and KIPR in the present study. Furthermore, the prognosis analysis indicated that high PANX1 expression was associated with poor outcomes in PAAD, LUAD, and KIPR. Therefore, our findings corroborate earlier research.

Interestingly, this study found inconsistent results in the relationship between immune cells infiltration and PANX1 using different approaches. The reasons for these disparities in findings are unclear. The following reasons may have contributed to this inconsistency. First, while assays such as flow cytometry, immunohistochemistry staining, or single-cell sequencing can estimate the immune cell status within a tumor sample, each has limitations that prevent them from being widely applicable. Therefore, computational methods were utilized to evaluate the immune-cell composition from bulk RNA-sequencing data [85]. As a result, there were some variations between the computer-based algorithms and the actual situation. Second, tumor immune cell infiltration mechanisms are complex and are inevitably influenced by other factors such as intratumoral heterogeneity and small sample size. Third, these methods are based on different algorithms such as marker gene-based and deconvolution-based algorithms, and each has its advantages and disadvantages.

Given that early data suggested PANX1 may predict poor prognosis and play essential roles in immune cell infiltration, we performed enrichment analysis of genes co-expressed with PANX1 in PAAD. GO findings showed that PANX1 was closely associated with leukocyte cell-cell adhesion and endoplasmic reticulum lumen in PAAD. These findings were consistent with the ones provided above. PANX1 was also found to be involved in PAAD immune cell recruitment as well as antigen processing and presentation. Moreover, KEGG pathway analysis also revealed that PANX1 was involved in ECM-receptor interactions and focal adhesion pathways. The findings might imply that PANX1 regulates ATP release and hence plays an essential role in tumor cells’ environment interaction.

The present study, however, has several limitations. Firstly, the study into the role of PANX1 in cancers relied on publicly available databases, and the predicted results were not validated by experimental methods. Therefore, these predicted results need to be validated in future studies. Secondly, the small sample size of some of the individual tumors in the databases may contribute to insufficient statistical power. Therefore, large sample sizes are required to validate the findings. Thirdly, while this study has shown certain aspects of immune cell infiltration in PAAD, the exact mechanism is still unknown. More experiments are needed to validate the predicted results and investigate the underlying molecular mechanisms.

In conclusion, the present study has revealed that PANX1 overexpression is correlated with poor prognosis and increased immune infiltration of CAF, macrophage, neutrophil, and myeloid dendritic cells in multiple cancers, particularly PAAD Furthermore, PANX1 expression is closely associated with immunomodulators such as CD274, IL10, CD276, CD80, and IL2RA. This study, on the other hand, is based on bioinformatics analysis and needs experimental validation. Therefore, further prospective studies are needed to validate the prognostic value of PANX1 and investigate underlying molecular mechanisms in tumor immunity.
